# Biography Contemporary Physicians of Texas

**Published:** 1889-10

**Authors:** 


THE BIOGRAPHY OF CONTEMPORARY PHYSICIANS OF TEXAS, now being compiled by the editor of this JOURNAL, will contain: The opening chapter, "Reminiscenses of Practice and Practitioners of Texas in Early Days," by Dr. T. J. Heard,—perhaps the oldest living practitioner in Texas. He begun in Washington county in 1837!—"A Complete History of the Organization and Development of the Texas State Medical Association," by Dr. R. H. L. Bibb, now of Saltillo, Mex.—"The Life and Character of Ashbel Smith, Statesman, Soldier, Surgeon," (with portrait), by James D. Lynch, Esq., of Miss.—The biography of a large number of leading physicians of the Lone Star State, including all but four of the presidents of the State Association, together with the portraits of thirty of the best known physicians in Texas. It will also contain a roster of a large number of practicing physicians whose biography in full does not appear in the work. Altogether this will make a most valuable work, and those who have not subscribed, should lose no time in doing so. It will be sold only by subscription. Agents are now in the field, and it is hoped to bring the work to a speedy completion.

---

ELMENDORF, Texas, Aug, 9, 1880.

*Dios Chemical Co., St. Louis, Mo:*

GENTLEMEN:—In regard to my experience with "Dioviburnia," I must say it is a splendid medicine and is just the thing recommended. No physician should be without it. Yours truly,

DR. LYMAN L. WHITAKER.

